# Childhood diabetes mellitus and early-onset kidney diseases later in life: a nationwide population-based matched cohort study

**DOI:** 10.1186/s12916-022-02634-4

**Published:** 2022-11-08

**Authors:** Jiahong Sun, Ce Wang, Min Zhao, Priscilla M. Y. Lee, Bo Xi, Yongfu Yu, Jiong Li

**Affiliations:** 1 Department of Epidemiology, School of Public Health, Qilu Hospital, Cheeloo College of Medicine, Shandong University, 44 Wen Hua Xi Road, Jinan, 250012 Shandong China; 2 Department of Biostatistics, School of Public Health, and The Key Laboratory of Public Health Safety of Ministry of Education, Fudan University, 130 Dong’an, Shanghai, 200032 China; 3 Department of Nutrition and Food Hygiene, Cheeloo College of Medicine, Shandong University, Jinan, Shandong China; 4 Department of Clinical Medicine-Department of Clinical Epidemiology, Aarhus University Hospital, Aarhus, Denmark; 5 Shanghai Institute of Infectious Disease and Biosecurity, Shanghai, China

**Keywords:** Diabetes mellitus, Kidney disease, Childhood, Adulthood

## Abstract

**Background:**

The empirical evidence remains inconclusive for an association between diabetes mellitus (DM) in children and early-onset kidney disease later in life, and little is known about the effects of DM types (i.e., type 1 diabetes [T1DM] and type 2 diabetes [T2DM]) in childhood on type-specific kidney diseases. We aimed to evaluate the association of childhood DM with overall and type-specific early-onset kidney diseases later in life.

**Methods:**

The population-based matched cohort study included 9356 individuals with DM (T1DM: 8470, T2DM: 886) diagnosed in childhood (< 18 years) who were born between 1977 and 2016, and 93,560 individuals without DM matched on sex and year of birth in Denmark. The main outcomes were overall and type-specific early-onset kidney diseases. The follow-up period of all included participants was from the date of DM diagnosis in the exposure group until the first diagnosis of kidney disease, emigration, or 31 December 2018, whichever came first.

**Results:**

During a median follow-up of 13 years, children with DM had a 154% increased risk of early-onset kidney diseases than children without DM (adjusted hazard ratios 2.54, 95% confidence intervals 2.38–2.72), and T1DM (2.48, 2.31–2.67) and T2DM (2.75, 2.28–3.31) showed similar results. Children with DM also had a higher risk of multiple specific kidney diseases including glomerular diseases, renal tubulo-interstitial diseases, renal failure, and urolithiasis. The risks of type-specific kidney diseases including glomerular diseases and renal failure tended to be higher for children with T2DM (glomerular diseases: 5.84, 3.69–9.24; renal failure: 14.77, 8.53–25.59) than those with T1DM (glomerular diseases: 3.14, 2.57–3.83; renal failure: 8.24, 6.66–10.20).

**Conclusions:**

Children with DM had a higher increased risk of early-onset overall and specific kidney diseases later in life. Early prevention and treatment of both T1DM and T2DM in childhood may significantly reduce the risk of kidney diseases later in life.

**Supplementary Information:**

The online version contains supplementary material available at 10.1186/s12916-022-02634-4.

## Background

Diabetes mellitus (DM) is one of the leading chronic medical disorders among children and adolescents [1]. The prevalence of type 1 diabetes mellitus (T1DM) and type 2 diabetes mellitus (T2DM) during childhood has been increasing worldwide [2–4]. An annual increase of 3.4% in the prevalence of T1DM between 1989 and 2013 was reported among European children under 14 years [3]. In the USA, the prevalence of T2DM among those aged < 19 years increased from 1.48 per 1000 youths in 2001 to 2.15 in 2017 [5]. In Denmark, the prevalence of T1DM has been increasing in childhood from ~ 0.20 per 1000 person-years in 1996 to ~ 0.35 per 1000 person-years in 2016 [6]. Although the prevalence of T2DM among Danish youth in 2014 was 0.6 per 100,000 inhabitants, T2DM was highly associated with overweight and obesity which has a significant increase in Danish youth [7, 8]. DM in children and adolescents poses a major public health burden and clinical challenges to pediatric and adult DM services [1, 9].

Youth-onset T2DM may cause longer exposure to the adverse effects of hyperglycemia than DM diagnosed in adulthood, thus leading to higher odds of progression to short-term and long-term detrimental complications, such as kidney lesions and cardiovascular diseases [10, 11]. Although several studies have suggested an association between DM (T1DM or T2DM) in children and early-onset kidney disease later in life, the empirical evidence remains inconclusive due to a relatively small number of DM cases, the representation of participants only recruited from diabetes centers, cross-sectional study design, and crude definition of nephropathy defined only according to the presence of microalbuminuria [12–20]. Besides, limited research on type-specific kidney diseases mainly focused on renal failure [15] and several studies have addressed end-stage renal disease in particular [19, 21–27]. However, the findings on the association between ages of diagnosis of DM (5–9 years vs. 10–14 years) and rate of end-stage renal disease were inconsistent [19, 23, 24]. Moreover, little is known about the effects of DM types in childhood on other type-specific kidney diseases, such as glomerular diseases and renal tubulo-interstitial diseases later in life, particularly taking into consideration the age of DM diagnosis, sex, and duration of DM. A better understanding of such association is imperative to prevent and control subsequent adverse kidney complications.

In this large-scale population-based cohort study, we aimed to assess the associations of childhood T1DM and T2DM with subsequent overall and type-specific early-onset kidney diseases including glomerular diseases, renal tubulo-interstitial diseases, renal failure, urolithiasis, injury of kidney, and other disorders of kidney and ureter, taking into consideration sex, age at diagnosis of DM, and duration of DM.

## Methods

### Study population and procedures

The unique personal identification number in the Danish Civil Registration System was used to accurately merge individual data from all national registers in Denmark [28]. A total of 102,916 participants aged 0–17 years born between January 1977 and December 2016 were included in this study, i.e., 9356 children with DM (8470 for T1DM and 886 for T2DM) from the Danish National Patient Register and 93,560 randomly selected children without DM (a ratio of 1:10 individually matched by sex and birth year) from the general population. Additional file 1: Fig. S1 shows the flow chart of inclusion and exclusion of study participants. The study was approved by the Data Protection Agency (record number 2013–41-2569). By Danish law, no informed consent is required for a register-based study of anonymized data.

We performed sibling comparison analyses to control for the influence of unmeasured familial and shared genetic characteristics [29, 30]. A sub-sample of 6908 families including 17,315 siblings born to the same mother discordant for both DM and subsequent kidney diseases contributed to the effect estimate. The date of diagnosis of siblings in the DM exposure group was used as the start date for both groups. The follow-up period of all included participants was from the date of DM diagnosis in the exposure group until the first diagnosis of kidney disease, emigration, or 31 December 2018, whichever came first.

### Exposure

Data on DM were from the Danish National Patient Registry, the Danish National Diabetes Register, and the Danish National Prescription Registry. We defined T1DM and T2DM as any first inpatient or outpatient visit with the diagnosis based on the International Classification of Disease (ICD) codes and prescription of anti-diabetic medicine by Anatomical Therapeutic Chemical (ATC) classification codes (T1DM: ICD-10 E10-E10.9, O24.0; ICD-8 249, ATC A10A; T2DM: ICD-10 E11-E119, O24.1; ICD-8 250, ATC A10B) (Additional file 1: Table S1). If one was diagnosed with both T1DM and T2DM, he/she was classified as the first type diagnosed DM. It should be noted that 91.0% of children with diabetes (T1DM or T2DM) had been mainly treated with insulin and its analogs, biguanides, and other drugs. Among all treated pediatric patients with T1DM, the proportions were 99.0% for the use of insulin and its analogs, 0.4% for biguanides, and 0.6% for other drugs. Among all treated pediatric patients with T2DM, the proportions were 43.6% for the use of insulin and its analogs, 50.4% for biguanides, and 6.0% for other drugs.

### Outcome of interest

Early-onset kidney disease in childhood or adulthood was identified at the first hospital diagnosis of glomerular diseases, renal tubulo-interstitial diseases, renal failure, urolithiasis, injury of the kidney, and other disorders of the kidney and ureter based on the Danish National Patient Register by using ICD-8 and ICD-10 codes (Additional file 1: Table S1).

### Covariates

Potential covariates used for adjustment included birthweight category (low, high, normal, or unknown), preterm birth (yes vs. no), parity (1, 2, or ≥ 3), singleton status (yes vs. no), residence (Copenhagen, cities with 100,000 or more inhabitants, or others), maternal marital status (unmarried, married, or unknown), maternal and paternal education levels (0–9 years, 10–14 years, ≥ 15 years, or unknown), and maternal and paternal history of DM (yes vs. no).

### Statistical analyses

Continuous variables were presented as mean ± standard deviation (SD) or median with interquartile range (P25–P75) and mean rank, and categorical variables were presented as frequency (percentage). Considering non-kidney disease deaths as the competing event, competing risk analysis was used to calculate the cumulative incidence between children with and without DM. Stratified Cox proportional regression analysis with inverse probability of treatment weighting was used to estimate the associations of childhood TIDM/T2DM with kidney diseases later in life after adjustment for the aforementioned covariates. We performed subgroup analyses stratified by sex, child age at diagnosis of DM (0–5, 6–12, and 13–17 years), diabetic duration (0–10, 11–20, and ≥ 21 years), calendar periods (1977–1985 vs. 1986–2016), and DM complications (≥ 1 complications vs. 0 complications). We performed sensitivity analyses to examine these associations including (1) the use of sibling sub-cohort design with stratified Cox proportional regression analyses to take into account the influence of unmeasured genetic and environmental characteristics [29], (2) the exclusion of 1-year, 3-year, and 5-year duration of DM from exposed the group to examine the potential detection bias of DM [18]; (3) the exclusion of DM patients in childhood without the use of hypoglycemic drugs (9.0%) to examine whether this therapy influenced the association; and (4) further adjustment for the use of nephroprotective therapy to examine whether this variable influenced the association. We used multiple imputation procedures to impute missing values of all covariates of interest for final data analyses. All analyses were performed using SAS version 9.4. A two-sided *p*-value < 0.05 was considered statistically significant.

## Results

Table 1 shows the baseline characteristics of participants by DM type. Children with DM were more likely to have low and high birth weight, preterm birth, lower maternal and paternal educational level, and parental history of DM before childbirth compared with children without DM. Similar results were found for most of these variables in children with T2DM compared with those with T1DM.

**Table 1:** Baseline characteristics of included participants by diabetes status

	Normal	DM	*P*-value (DM vs. normal)	T1DM	T2DM	*P*-value (T2DM vs. T1DM)
Overall	93,560	9356		8470	886	
Birthweight, g, mean ± SD						
Mean ± SD	3448.8 ± 632.8	3447.9 ± 669.9	0.900	3462.4 ± 667.3	3309.5 ± 679.3	< 0.001
Median (P25–P75)	3500.0 (3100.0–3830.0)	3500.0 (3100.0–3850.0)	0.709	3500.0 (3100.0–3850.0)	3350.0 (3000.0–3740.0)	
Mean rank	51,456.2	51,481.5		4740.1	4089.9	
Birth weight categories, *n* (%)			0.001			< 0.001
Normal	72,166 (77.1)	7100 (75.9)		6419 (75.8)	681 (76.9)	
Low	4746 (5.1)	548 (5.9)		469 (5.5)	79 (8.9)	
High	15,928 (17.0)	1653 (17.7)		1529 (18.1)	124 (14.0)	
Unknown	720 (0.8)	55 (0.5)		53 (0.6)	2 (0.2)	
Gestational age, weeks						
Mean ± SD	39.4 ± 2.0	39.2 ± 2.2	< 0.001	39.3 ± 2.1	39.0 ± 2.3	0.005
Median (P25–P75)	40.0 (39.0–41.0)	40.0 (38.0–40.0)	< 0.001	40.0 (38.0–40.0)	40.0 (38.0–40.0)	0.033
Mean rank	51,729.4	48,749.6		4677.9	4684.0	
Preterm birth, *n* (%)			< 0.001			< 0.001
Yes	5034 (5.4)	637 (6.8)		556 (6.6)	81 (9.1)	
No	83,211 (88.9)	8203 (87.7)		7479 (88.3)	724 (81.8)	
Unknown	5315 (5.7)	516 (5.5)		435 (5.1)	81 (9.1)	
Age at follow-up, years						
Mean ± SD	23.79 ± 9.28	23.49 ± 9.17	< 0.003	22.94 ± 9.16	28.80 ± 7.31	< 0.001
Median (P25–P75)	23.0 (17.0–31.0)	23.0 (17.0–30.0)	0.005	22.0 (16.0–29.0)	29.0 (24.0–34.0)	< 0.001
Mean rank	51,541.5	50,628.2		4508.5	6303.8	
Age at baseline, *n* (%)			1.000			
0–5 years	21,840 (23.3)	2184 (23.3)		2017 (23.8)	167 (18.8)	
6–12 years	43,210 (46.2)	4321 (46.2)		4083 (48.2)	238 (26.9)	
13–17 years	28,510 (30.5)	2851 (30.5)		2370 (28.0)	481 (54.3)	
Duration of diabetes, years, median (P25–P75)	–	12.26 (6.23–19.59)		11.99 (5.87–19.20)	14.57 (8.95–26.27)	< 0.001
Mean rank	–	–		4582.1	5600.2	
Calendar year, *n* (%)			1.000			< 0.001
1977–1985	18,380 (19.6)	1838 (19.6)		1544 (18.2)	294 (33.2)	
1986–1995	30,650 (32.8)	3065 (32.8)		2638 (31.1)	427 (48.2)	
1996–2016	44,530 (47.6)	4453 (47.6)		4288 (51.7)	165 (18.6)	
Sex, *n* (%)			1.000			< 0.001
Male	48,850 (52.2)	4885 (52.2)		4528 (53.5)	357 (40.4)	
Female	44,710 (47.8)	4471 (47.8)		3942 (46.5)	529 (59.7)	
Parity, *n* (%)			0.134			0.233
1	41,463 (44.3)	4066 (43.5)		3675 (43.4)	391 (44.1)	
2	35,013 (37.4)	3512 (37.5)		3200 (37.8)	312 (35.2)	
≥ 3	17,084 (18.3)	1778 (19.0)		1595 (18.8)	183 (20.7)	
Singleton status, *n* (%)			0.641			0.051
Yes	90,622 (96.9)	9071 (97.0)		8202 (96.8)	869 (98.1)	
No	2938 (3.1)	285 (3.0)		268 (3.2)	17 (1.9)	
Paternal education level, *n* (%)			< 0.001			< 0.001
0–9 years	21,758 (23.3)	2202 (24.6)		2017 (23.8)	285 (32.2)	
10–14 years	49,820 (53.2)	5044 (53.9)		4583 (54.1)	461 (52.0)	
≥ 15 years	18,249 (19.5)	1697 (18.1)		1596 (18.8)	101 (11.4)	
Unknown	3733 (4.0)	313 (3.4)		274 (3.2)	39 (4.4)	
Maternal education level, *n* (%)			< 0.001			< 0.001
0–9 years	26,556 (28.4)	2790 (29.8)		2408 (28.4)	382 (43.1)	
10–14 years	41,646 (44.5)	4195 (44.8)		3848 (45.4)	347 (39.2)	
≥ 15 years	24,217 (25.9)	2287 (24.4)		2142 (25.3)	145 (16.4)	
Unknown	1141 (1.2)	89 (0.9)		72 (0.9)	12 (1.4)	
Residence, *n* (%)			0.004			< 0.001
Copenhagen	9665 (10.3)	877 (9.4)		759 (9.0)	118 (13.3)	
Cities with 100,000 or more inhabitants	11,903 (12.7)	1149 (12.3)		1052 (12.4)	97 (10.9)	
Others	71,992 (76.9)	7330 (78.3)		6659 (78.6)	671 (75.8)	
Maternal marital status, *n* (%)			0.089			0.002
Unmarried	40,985 (43.8)	4054 (43.3)		3714 (43.8)	340 (38.4)	
Married	52,535 (56.2)	5302 (56.7)		4756 (56.2)	546 (61.6)	
Unknown	40 (0.0)	0 (0.0)		0	0	
Maternal history of diabetes before childbirth, *n* (%)			< 0.001			< 0.001
Yes	1412 (1.5)	449 (4.8)		376 (4.4)	73 (8.2)	
No	93,048 (98.5)	8907 (95.2)		8094 (95.6)	813 (91.8)	
Paternal history of diabetes before childbirth, *n* (%)			< 0.001			0.008
Yes	558 (0.6)	423 (4.5)		399 (4.7)	24 (2.7)	
No	93,002 (99.4)	8933 (95.5)		8071 (95.3)	862 (97.3)	
Maternal age at childbirth (years)			0.572			< 0.001
< 20	2333 (2.5)	217 (2.3)		181 (2.1)	36 (4.1)	
20–24	17,373 (18.6)	1700 (18.2)		1481 (17.5)	219 (24.7)	
25–29	34,975 (37.4)	3501 (37.4)		3171 (37.4)	330 (37.3)	
30–34	27,325 (29.2)	2790 (29.8)		2584 (30.5)	206 (23.3)	
≥ 35	11,554 (12.4)	1148 (12.3)		1053 (12.4)	95 (10.7)	

*T1DM*, type 1 diabetes mellitus; *T2DM*, type 2 diabetes mellitus

### Associations of childhood DM with overall and type-specific early-onset kidney diseases

**Fig. 1:**
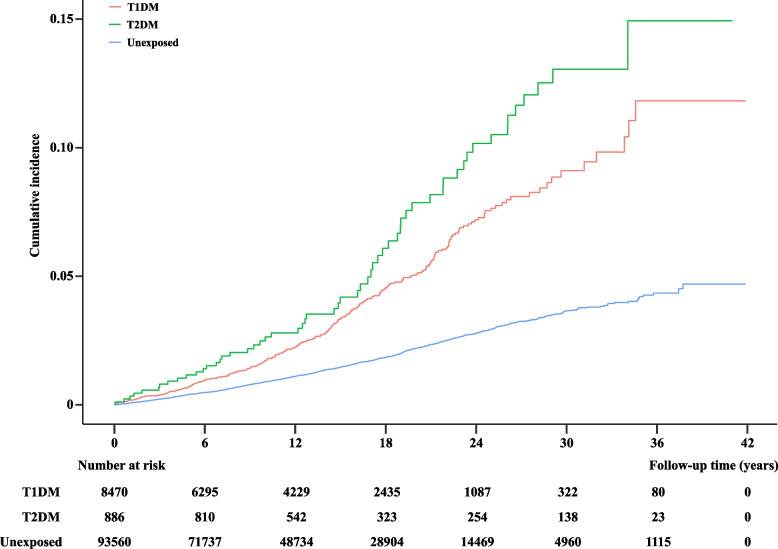
Cumulative incidence of overall early-onset kidney diseases later in life among children divided into three groups (without DM, with T1DM, and with T2DM). DM, diabetes mellitus; T1DM, type 1 diabetes mellitus; T2DM, type 2 diabetes mellitus

During a median follow-up of 13 years, 340 children with DM (T1DM: 283, T2DM: 57) and 1376 children without DM were diagnosed with kidney disease. The cumulative incidence of any kidney diseases among individuals with DM was higher than those without DM, and the cumulative incidence seemed slightly higher in T2DM than in T1DM (Fig. 1). Children with DM had a 154% increased risk of early-onset kidney diseases, compared with children without DM (adjusted hazard ratios [aHR] 2.54, 95% confidence intervals 2.38–2.72). Both T1DM (2.48, 2.31–2.67) and T2DM (2.75, 2.28–3.31) were associated with an increased risk of overall kidney diseases. The risks for type-specific kidney diseases, in particular for glomerular disease (aHR = 3.56, 95% CI = 2.97–4.27), renal tubulo-interstitial diseases (aHR = 2.74, 95% CI = 2.48–3.02), renal failure (aHR = 9.13, 95% CI = 7.49–11.14), and urolithiasis (aHR = 1.39, 95% CI = 1.20–1.61) among children with DM, were also increased (Table 2). Similar patterns were found for T1DM and T2DM while the magnitude of associations seemed slightly stronger in children with T2DM than in those with T1DM (Table 2). Analyses stratified by sex showed similar results for any kidney diseases, glomerular disease, renal tubulo-interstitial disease, and renal failure. We observed an increased risk of urolithiasis among females with DM (1.93, 1.61–2.31) but not among males with DM (0.92, 0.71–1.19) (Additional file 1: Table S2).

**Table 2:** Adjusted hazard ratios (95% confidence intervals) of overall and type-specific early-onset kidney diseases later in life for children with diabetes

Type of kidney disease	DM				T1DM				T2DM			
	No. (%) of kidney disease in exposed/unexposed groups	Rate per 1000 person-years in exposed/unexposed groups	Crude HR (95% CI)	Adjusted HR (95% CI)	No. (%) of kidney disease in exposed/unexposed groups	Rate per 1000 person-years in exposed/unexposed groups	Crude HR (95% CI)	Adjusted HR (95% CI)	No. (%) of kidney disease in exposed/unexposed group	Rate per 1000 person-years in exposed/unexposed groups	Crude HR (95% CI)	Adjusted HR (95% CI)
Any kidney disease	340(3.63)/1376(1.47)	26.93/10.66	2.54 (2.25–2.86)	2.54 (2.38–2.72)	283(3.34)/1176(1.39)	25.45/10.36	2.47 (2.17–2.81)	2.48 (2.31–2.67)	57(6.43)/200(2.26)	37.87/12.88	2.96 (2.20–3.98)	2.75 (2.28–3.31)
Glomerular diseases	57(0.61)/161(0.17)	4.51/1.25	3.58 (2.64–4.84)	3.56 (2.97–4.27)	42(0.50)/135(0.16)	3.77/1.19	3.12 (2.20–4.41)	3.14 (2.57–3.83)	15(1.69)/26(0.29)	9.96/1.67	6.11 (3.21–11.65)	5.84 (3.69–9.24)
Renal tubulo-interstitial diseases	166(1.77)/619(0.66)	13.14/4.80	2.75 (2.32–3.27)	2.74 (2.48–3.02)	147(1.74)/538(0.64)	13.21/4.74	2.81 (2.34–3.38)	2.83 (2.55–3.14)	19(2.14)/81(0.91)	12.62/5.22	2.34 (1.42–3.86)	2.00 (1.46–2.74)
Renal failure	100(1.07)/115(0.12)	7.91/0.89	9.16 (6.97–12.03)	9.13 (7.49–11.14)	78(0.92)/98(0.12)	7.01/0.86	8.23 (6.09–11.13)	8.24 (6.66–10.20)	22(2.48)/17(0.19)	14.58/1.09	15.26 (7.81–29.83)	14.77 (8.53–25.59)
Urolithiasis	65(0.69)/491(0.52)	5.15/3.80	1.37 (1.06–1.78)	1.39 (1.20–1.61)	50(0.59)/409(0.48)	4.49/3.60	1.26 (0.94–1.69)	1.27 (1.07–1.50)	15(1.69)/82(0.93)	9.96/5.28	1.96 (1.12–3.40)	1.94 (1.40–2.69)
Injury of kidney	8(0.09)/72(0.08)	0.63/0.56	1.15 (0.55–2.38)	1.14 (0.75–1.75)	8(0.09)/65(0.08)	0.72/0.57	1.28 (0.61–2.66)	1.28 (0.85–1.95)	–	–	–	–
Other disorders of kidney and ureter	41(0.44)/89(0.10)	3.25/0.69	4.71 (3.25–6.83)	4.80 (3.80–6.05)	32(0.38)/74(0.09)	2.88/0.65	4.46 (2.94–6.78)	4.57 (3.54–5.90)	9(1.02)/15(0.17)	5.98/0.97	5.87 (2.57–13.41)	5.42 (2.99–9.81)

Cox proportional regression analyses with inverse probability of treatment weighting were adjusted for birthweight category, preterm birth, parity, singleton status, maternal residence, maternal marital status, maternal and paternal education level, and maternal and paternal history of diabetes

“–” indicates data are unavailable

*HR*, hazard ratio; *CI*, confidence interval; *DM*, diabetes mellitus; *T1DM*, type 1 diabetes mellitus; *T2DM*, type 2 diabetes mellitus

### Associations of childhood DM with early-onset kidney diseases by calendar years, age of DM diagnosis, DM types, DM duration, and DM complications

**Fig. 2:**
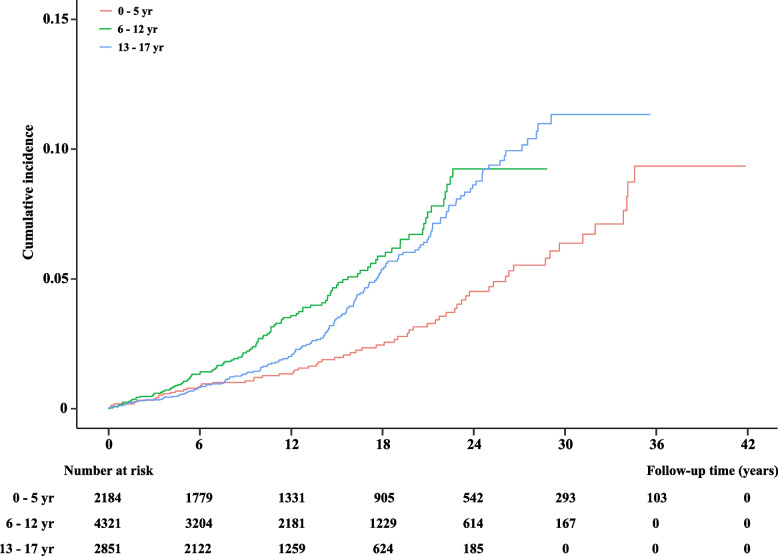
Cumulative incidence of overall early-onset kidney diseases later in life among children by age of diagnosis of diabetes mellitus (0–5, 6–12, and 13–17 years)

The results for DM and overall and specific types of early-onset kidney diseases were stable during different calendar periods (1977–1985 vs. 1986–2016) (Additional file 1: Table S3). When we stratified the age of DM diagnosis, older exposed children at 6–12 years or 13–17 years were more likely to have a higher rate of kidney disease than younger children at 0–5 years (Fig. 2 and Additional file 1: Table S4). Similar results were found according to DM types (Additional file 1: Fig. S2 and Fig. S3). We found that children with longer DM duration (10–20 years and ≥ 21 years vs. 0–10 years) were at a higher increased risk of kidney diseases (Additional file 1: Table S5). Children with DM in combination with more than one diabetic complications (vs. without diabetic complication) tended to have a higher risk of kidney diseases later in life (Additional file 1: Table S6).

### Sensitivity analyses

The results from the sibling cohort were similar to the main results from the general population cohort (Additional file 1: Table S7). After excluding children with follow-up after a diagnosis of DM less than 1 year, 3 year, and 5 year, the results also remained stable (Additional file 1: Table S8). After further excluding DM patients in childhood without the use of hypoglycemic drugs also did not substantially change the findings (Additional file 1: Table S9). After further adjustment for the use of nephroprotective therapy, the results also remained consistent (Additional file 1: Table S10).

## Discussion

Our study showed that children with T1DM or T2DM had an increased risk of overall and type-specific early-onset kidney diseases than those without DM. Older children who received a diagnosis of DM at 6–17 years (vs. younger children with DM diagnosed at 0–5 years) and children with longer DM duration (vs. shorter DM duration) were more likely to have overall and type-specific early-onset kidney diseases later in life. Females with DM were more likely to have urolithiasis, whereas males were not.

Several previous studies have reported that the overall risks of kidney diseases increased in young people with DM [15, 17, 31]. However, little is known about the association of DM with type-specific kidney diseases later in life. A cohort study based on 342 Canadian youths showed that DM was associated with an increased risk of renal failure compared with non-DM [15]. However, only a small number of individuals (365 with T1DM and 56 with T2DM) were followed up for more than 10 years. Although several previous studies in Norway, Sweden, and Finland showed that children or young people with DM had an increased risk of end-stage renal disease [19, 23–25], evidence is scarce on the cumulative risks of other kidney diseases for young people with DM. In this study, we used nationwide data in Denmark and confirmed that children with DM had an increased overall risk of kidney diseases, and we firstly reported that children with either type of DM had an increased risk of type-specific kidney diseases including glomerular disease, renal tubulo-interstitial disease, renal failure, and urolithiasis. Exposure to hyperglycemia could drive the loss of kidney function, influencing both renal tubulointerstitial and glomerular filtration barriers through increased oxidative stress, cell apoptosis, tissue fibrosis, and inflammation [32]. Our findings support that it is imperative to achieve and maintain glycemic control in youths [33] to prevent overall and type-specific early-onset kidney outcomes later in life. It is documented that improved glucose control may be useful for delaying the onset and progression of early complications of DM (including T1DM and T2DM) [34, 35]. Parental involvement in school and social settings, anticipation of poorer adherence and glucose control in youth with DM, a concern of depression symptoms, and appropriate health care services may help manage glucose more optimally [36].

The study based on 4555 children and adolescents from a young diabetes registry showed that the incidence rate of nephropathy per 1000 person-years increased with the duration of T1DM and T2DM [31]. However, this study was only based on a tertiary care private DM center in India, which might not be generalized to other populations [31]. Moreover, the effects of youth-onset DM on type-specific kidney diseases were not investigated. Two previous studies showed that both young and old people with a longer duration of DM had an increased risk of end-stage kidney disease [19, 27], but data on other type-specific kidney diseases are lacking. In this study, we found that the risks of kidney diseases overall and specific types (including renal failure, glomerular diseases, renal tubulo-interstitial disease, and urolithiasis) increased with the DM duration. Besides, we found that renal tubulo-interstitial disease and renal failure may occur with a short DM duration of fewer than 10 years, which suggest that it is important to keep healthy blood glucose level earlier to prevent these kidney diseases later in life.

Interestingly, similar to previous studies on the association between T2DM and long-term end-stage kidney disease in Canadian and Australian young adults [27, 37], we found that DM diagnosed at 0–5 years afforded lower cumulative incidence of all kidney diseases than DM diagnosed at 6–12 years and 13–17 years, independent of types of DM. Data from the Swedish Childhood Diabetes Register showed that the cumulative incidence of end-stage kidney disease for T1DM diagnosed at 0–9 years was lower than that diagnosed at 10–19 years [19]. However, this study did not compare the rate of end-stage kidney disease between T1DM diagnosed at 0–5 years and that at 5–9 years. In Norwegian children, the cumulative incidence of end-stage kidney disease for T1DM diagnosed at 0–5 years and 5–9 years was lower than that diagnosed at 10–14 years [24], whereas in Finnish children, the cumulative incidence of end-stage kidney disease for T1DM diagnosed at 0–4 years was lower than that diagnosed at 5–9 years and 10–14 years [23]. Our findings on the age of diagnosis of DM and cumulative incidence of all kidney diseases were similar to the latter one. The higher cumulative incidence of kidney diseases for diagnosed DM at 6–17 years compared with diagnosis at 0–5 years might be due to that sexual maturation caused by psychological and endocrine factors at puberty appears to accelerate the progress of DM, reflux nephropathy, and posterior urethral valves [38]. Moreover, the large psychological changes in puberty might influence the adherence to DM treatment [39], which poses a challenge to achieving good DM care for youths with DM. Besides, other risk factors including a rapid increase in body weight and blood pressure and altered endocrine during the puberty period also influence the development of chronic kidney disease [38]. In general, detecting DM in early childhood might be important for physicians helping them improve glycemic control, increase monitoring intensity, and delay long-term DM complications. It has been demonstrated that DM diagnosed at a young age might contribute to a delay in the development of end-stage kidney disease [23, 24].

Although several studies reported a higher or similar incidence of renal complications in T1DM than that in T2DM [12, 40, 41], a large number of emerging studies have shown that youths with T2DM had a higher burden of renal complications (especially end-stage kidney diseases or renal failure) and poor prognosis than those with T1DM [15, 17, 31, 40, 42]. The discrepancy of previous studies might be due to the differences in age and ethnicity/race of study participants, study sample size, the definition of DM, duration of follow-up years, and various renal complications (such as kidney risk profile, renal failure, and diabetic kidney diseases). In this study, we found that children with T1DM or T2DM had a similar increased overall risk of kidney disease, renal tubulo-interstitial disease, and urolithiasis compared with those without DM. The similarity in the risks of type-specific kidney diseases due to T1DM and T2DM implies shared pathological mechanisms between both types of DM in the development of kidney diseases [32]. However, the odds of glomerular disease and kidney failure among children with T2DM were nearly twice the odds among those with T1DM. Our findings might partly explain the discrepancy of previous findings. Although the potential mechanisms have not been elucidated, obesity, which causes T2DM, might play an important role in the formation of these kidney diseases [43, 44]. Besides, it might be driven by other major risk factors such as hypertension and dyslipidemia [45].

We found a similar risk of glomerular disease for youth-onset DM among both males and females, which was in line with the finding on the association between childhood-onset T1DM and end-stage kidney disease in a nationwide study in Sweden [25], but inconsistent with the finding of a markedly higher risk of end-stage kidney disease for the onset of DM among Australian males at 10–29 years vs. females and among Swedish males at 20–34 years vs. females [25, 27]. We additionally observed that females with DM were more likely to have urolithiasis, whereas males were not. Previous evidence has indicated that pediatric nephrolithiasis was more common in females than in males [46]. These findings might be explained by the fact that females had more acquisition of bone mineralization driven by estrogen, which might cause the formation of urine chemistry and stone [47]. At the start of puberty, females had higher levels of urinary citrate than males [48]. These findings imply that sex plays a vital role in the development of urolithiasis among children with DM, and further studies are warranted to understand the mechanism. However, we could not assess the sex effect on the association between childhood DM types and type-specific kidney diseases due to limited cases when stratified by sex, which needs further exploration.

To our knowledge, this was the first nationwide prospective study that offered new insights into the incidence of type-specific kidney diseases later in life for children with T1DM or T2DM. Additionally, we performed sibling design to assess the influence of unmeasured genetic and environmental characteristics, which are difficult to adjust using a conventional cohort study design. Several limitations deserved consideration. First, the small numbers of some type-specific kidney outcomes precluded us from performing subgroup analyses. Second, although several confounders have been adjusted in this study, confounding effects of some unadjusted factors cannot be ruled out, such as obesity status and lifestyles in childhood or adulthood. Third, the same code (ICD-8, 205) of T1DM and T2DM before 1986 might cause potential misclassification bias because children with T2DM who required insulin treatment might be misclassified as T1DM. However, our subgroup analyses stratified by calendar periods yielded a similar association between DM and the overall risk of kidney diseases between 1977–1985 and 1986–2016. Fourth, the use of ICD codes might underestimate the incidence of chronic kidney disease in administrative data because of inadequate documentation in discharge summaries and/or inaccurate coding practice [49]. However, we used both Danish-modified ICD-10 codes, ICD-8 codes, and ATC codes to capture kidney diseases. Fifth, we only examined the risk of kidney diseases in childhood and early adulthood because of the short follow-up duration (median: 13 years), and limited follow-up duration in our study might omit young children born in 1996–2016 who had a high risk of T2DM in adolescence. Future well-designed cohort studies with longer follow-up duration are warranted.

## Conclusions

In conclusion, we found that childhood DM (both T1DM and T2DM) could lead to an increased risk of early-onset kidney diseases overall and a number of specific types, such as glomerular disease, renal tubulo-interstitial disease, and renal failure later in life. Older children who received a diagnosis of DM during later childhood were at a higher risk of developing kidney diseases compared with those during early childhood. Youth-onset DM with a longer duration was associated with a higher increased risk of overall and type-specific early-onset kidney diseases later in life. These findings highlight the significance of early detection and prevention of T1DM and T2DM among children at high risk because of prolonged exposure and stress and the importance of strict control of DM in the life course to reduce the high burden of kidney disease later in life.

## Supplementary Information

**Additional file 1: **Supplementary results.** Fig. S1.** Flow chartof inclusion/exclusion of participants in this study.** Fig. S2.** Cumulative incidence of overall early-onset kidneydiseases later in life among children by age of diagnosis of type 1 diabetesmellitus (0-5, 6-12, and 13-17 years). **Fig. S3.** Cumulative incidence of overall early-onset kidney diseases later inlife among children by age of diagnosis of type 2 diabetes mellitus (0-5, 6-12,and 13-17 years). **Table S1. **International Classification of Diseases codes for diabetes mellitus and kidneydisease.** Table S2.** Associations ofchildhood diabetes with overall and type-specific early-onset kidney diseaseslater in life stratified for sex. **TableS3.** Associations of childhood diabetes with overall and type-specificearly-onset kidney diseases later in life stratified for periods of birth. **Table S4.** Associations of childhooddiabetes with overall and type-specific early-onset kidney diseases later inlife stratified for diagnosed age. **TableS5.** Associations of childhood diabetes with overall and type-specificearly-onset kidney disease later in life stratified for diabetes duration. **Table S6.** Associations of childhooddiabetes with overall and type-specific early-onset kidney disease later inlife stratified for number of diabetic complications. **Table S7.** Associations of childhood diabetes with overall andtype-specific early-onset kidney diseases later in life using a sibling design.**Table S8.** Associations of childhooddiabetes with overall and type-specific early-onset kidney diseases later inlife after excluding participants with one, three, or five years of diabetesduration. **Table S9.** Associations ofchildhood diabetes with overall and type-specific early-onset kidney diseaselater in life after exclusion of those without the use of hypoglycemic drugs. **Table S10.** Associations of childhooddiabetes with overall and type-specific early-onset kidney diseases later inlife after further adjustment for the use of nephroprotective therapy.

## Data Availability

Data collected for this study and additional related documents will be available to others by contacting the corresponding author (Prof. Yongfu Yu, email: yu@fudan.edu.cn). Questions related to data availability should contact the senior author (Prof. Jiong Li, Email: jl@clin.au.dk ).

## Abbreviations

aHR: Adjusted hazard ratios
ATC: Anatomical Therapeutic Chemical
DM: Diabetes mellitus
ICD: International Classification of Disease
SD: Standard deviation
T1DM: Type 1 diabetes mellitus
T2DM: Type 2 diabetes mellitus

## References

[1] Cameron FJ, Wherrett DK (2015). Care of diabetes in children and adolescents: controversies, changes, and consensus. Lancet.

[2] Divers J, Mayer-Davis EJ, Lawrence JM, Isom S, Dabelea D, Dolan L (2020). Trends in incidence of type 1 and type 2 diabetes among youths - selected counties and Indian reservations United States 2002–2015. MMWR Morb Mortal Wkly Rep.

[3] Patterson CC, Harjutsalo V, Rosenbauer J, Neu A, Cinek O, Skrivarhaug T (2019). Trends and cyclical variation in the incidence of childhood type 1 diabetes in 26 European centres in the 25 year period 1989–2013: a multicentre prospective registration study. Diabetologia.

[4] Nadeau KJ, Anderson BJ, Berg EG, Chiang JL, Chou H, Copeland KC (2016). Youth-onset type 2 diabetes consensus report: current status, challenges, and priorities. Diabetes Care.

[5] Lawrence JM, Divers J, Isom S, Saydah S, Imperatore G, Pihoker C (2021). Trends in prevalence of type 1 and type 2 diabetes in children and adolescents in the US, 2001–2017. JAMA.

[6] Carstensen B, Ronn PF, Jorgensen ME. Prevalence, incidence and mortality of type 1 and type 2 diabetes in Denmark 1996–2016. BMJ Open Diabetes Res Care. 2020; 8(1). 10.1136/bmjdrc-2019-001071. 10.1136/bmjdrc-2019-001071 PMC7265004 32475839

[7] Oester IM, Kloppenborg JT, Olsen BS, Johannesen J (2016). Type 2 diabetes mellitus in Danish children and adolescents in 2014. Pediatr Diabetes.

[8] Rasmussen M, Damsgaard MT, Morgen CS, Kierkegaard L, Toftager M, Rosenwein SV (2020). Trends in social inequality in overweight and obesity among adolescents in Denmark 1998–2018. Int J Public Health.

[9] Viner R, White B, Christie D (2017). Type 2 diabetes in adolescents: a severe phenotype posing major clinical challenges and public health burden. Lancet.

[10] Looker HC, Pyle L, Vigers T, Severn C, Saulnier PJ, Najafian B (2022). Structural lesions on kidney biopsy in youth-onset and adult-onset type 2 diabetes. Diabetes Care.

[11] Zeitler P, Chou HS, Copeland KC, Geffner M (2015). Clinical trials in youth-onset type 2 diabetes: needs, barriers, and options. Curr Diab Rep.

[12] Tommerdahl KL, Baumgartner K, Schafer M, Bjornstad P, Melena I, Hegemann S (2021). Impact of obesity on measures of cardiovascular and kidney health in youth with type 1 diabetes as compared with youth with type 2 diabetes. Diabetes Care.

[13] Fang M, Echouffo-Tcheugui JB, Selvin E (2020). Burden of complications in U.S. adults with young-onset type 2 or type 1 diabetes. Diabetes Care.

[14] Wong J, Constantino M, Yue DK (2015). Morbidity and mortality in young-onset type 2 diabetes in comparison to type 1 diabetes: where are we now?. Curr Diab Rep.

[15] Dart AB, Sellers EA, Martens PJ, Rigatto C, Brownell MD, Dean HJ (2012). High burden of kidney disease in youth-onset type 2 diabetes. Diabetes Care.

[16] Amutha A, Anjana RM, Venkatesan U, Ranjani H, Unnikrishnan R, Narayan KMV (2017). Incidence of complications in young-onset diabetes: comparing type 2 with type 1 (the young diab study). Diabetes Res Clin Pract.

[17] Dabelea D, Stafford JM, Mayer-Davis EJ, D’Agostino R, Dolan L, Imperatore G (2017). Association of type 1 diabetes vs type 2 diabetes diagnosed during childhood and adolescence with complications during teenage years and young adulthood. JAMA.

[18] Dabelea D, Hamman RF, Knowler WC. Diabetes in Youth. In: Cowie CC, Casagrande SS, Menke A, Cissell MA, Eberhardt MS, Meigs JB, Gregg EW, Knowler WC, Barrett-Connor E, Becker DJ, Brancati FL, Boyko EJ, Herman WH, Howard BV, Narayan KMV, Rewers M, Fradkin JE, editors. Diabetes in America. 3rd ed. Bethesda (MD): National Institute of Diabetes and Digestive and Kidney Diseases (US); 2018. CHAPTER 15.

[19] Toppe C, Mollsten A, Waernbaum I, Schon S, Gudbjornsdottir S, Landin-Olsson M (2019). Decreasing cumulative incidence of end-stage renal disease in young patients with type 1 diabetes in Sweden: a 38-year prospective nationwide study. Diabetes Care.

[20] Constantino MI, Molyneaux L, Limacher-Gisler F, Al-Saeed A, Luo C, Wu T (2013). Long-term complications and mortality in young-onset diabetes: type 2 diabetes is more hazardous and lethal than type 1 diabetes. Diabetes Care.

[21] Luk AO, Lau ES, So WY, Ma RC, Kong AP, Ozaki R (2014). Prospective study on the incidences of cardiovascular-renal complications in Chinese patients with young-onset type 1 and type 2 diabetes. Diabetes Care.

[22] Pavkov ME, Bennett PH, Knowler WC, Krakoff J, Sievers ML, Nelson RG (2006). Effect of youth-onset type 2 diabetes mellitus on incidence of end-stage renal disease and mortality in young and middle-aged Pima Indians. JAMA.

[23] Helve J, Sund R, Arffman M, Harjutsalo V, Groop PH, Gronhagen-Riska C (2018). Incidence of end-stage renal disease in patients with type 1 diabetes. Diabetes Care.

[24] Gagnum V, Saeed M, Stene LC, Leivestad T, Joner G, Skrivarhaug T (2018). Low incidence of end-stage renal disease in childhood-onset type 1 diabetes followed for up to 42 years. Diabetes Care.

[25] Mollsten A, Svensson M, Waernbaum I, Berhan Y, Schon S, Nystrom L (2010). Cumulative risk, age at onset, and sex-specific differences for developing end-stage renal disease in young patients with type 1 diabetes: a nationwide population-based cohort study. Diabetes.

[26] Svensson M, Nystrom L, Schon S, Dahlquist G (2006). Age at onset of childhood-onset type 1 diabetes and the development of end-stage renal disease: a nationwide population-based study. Diabetes Care.

[27] Morton JI, Liew D, McDonald SP, Shaw JE, Magliano DJ (2020). The association between age of onset of type 2 diabetes and the long-term risk of end-stage kidney disease: a national registry study. Diabetes Care.

[28] Schmidt M, Schmidt SAJ, Adelborg K, Sundboll J, Laugesen K, Ehrenstein V (2019). The Danish health care system and epidemiological research: from health care contacts to database records. Clin Epidemiol.

[29] Yu Y, Arah OA, Liew Z, Cnattingius S, Olsen J, Sorensen HT (2019). Maternal diabetes during pregnancy and early onset of cardiovascular disease in offspring: population based cohort study with 40 years of follow-up. BMJ.

[30] Du J, Li J, Liu X, Liu H, Obel C, Shen H (2021). Association of maternal diabetes during pregnancy with high refractive error in offspring: a nationwide population-based cohort study. Diabetologia.

[31] Amutha A, Ranjit U, Anjana RM, Shanthi RC, Rajalakshmi R, Venkatesan U (2021). Clinical profile and incidence of microvascular complications of childhood and adolescent onset type 1 and type 2 diabetes seen at a tertiary diabetes center in India. Pediatr Diabetes.

[32] Ricciardi CA, Gnudi L (2021). Kidney disease in diabetes: from mechanisms to clinical presentation and treatment strategies. Metabolism.

[33] Zeitler P, Hirst K, Pyle L, Linder B, Copeland K, Today Study Group (2012). A clinical trial to maintain glycemic control in youth with type 2 diabetes. N Engl J Med..

[34] Shichiri M, Kishikawa H, Ohkubo Y, Wake N (2000). Long-term results of the Kumamoto study on optimal diabetes control in type 2 diabetic patients. Diabetes Care.

[35] Fullerton B, Jeitler K, Seitz M, Horvath K, Berghold A, Siebenhofer A (2014). Intensive glucose control versus conventional glucose control for type 1 diabetes mellitus. Cochrane Database Syst Rev.

[36] Anderson BJ, McKay SV (2011). Barriers to glycemic control in youth with type 1 diabetes and type 2 diabetes. Pediatr Diabetes.

[37] Jiang Y, Osgood N, Lim HJ, Stang MR, Dyck R (2014). Differential mortality and the excess burden of end-stage renal disease among First Nations people with diabetes mellitus: a competing-risks analysis. CMAJ.

[38] Lane PH (2005). Puberty and chronic kidney disease. Adv Chronic Kidney Dis.

[39] Morris AD, Boyle DI, McMahon AD, Greene SA, MacDonald TM, Newton RW (1997). Adherence to insulin treatment, glycaemic control, and ketoacidosis in insulin-dependent diabetes mellitus. The DARTS/MEMO collaboration. Diabetes audit and research in Tayside Scotland. Medicines monitoring unit. Lancet.

[40] Pleniceanu O, Twig G, Tzur D, Gruber N, Stern-Zimmer M, Afek A (2021). Kidney failure risk in type 1 vs. type 2 childhood-onset diabetes mellitus. Pediatr Nephrol.

[41] Bogdanovic R (2008). Diabetic nephropathy in children and adolescents. Pediatr Nephrol.

[42] Wang SY, Andrews CA, Herman WH, Gardner TW, Stein JD (2017). Incidence and risk factors for developing diabetic retinopathy among youths with type 1 or type 2 diabetes throughout the United States. Ophthalmology.

[43] D’Agati VD, Chagnac A, de Vries AP, Levi M, Porrini E, Herman-Edelstein M (2016). Obesity-related glomerulopathy: clinical and pathologic characteristics and pathogenesis. Nat Rev Nephrol.

[44] Nehus E, Mitsnefes M (2019). Childhood obesity and the metabolic syndrome. Pediatr Clin North Am.

[45] Magliano DJ, Sacre JW, Harding JL, Gregg EW, Zimmet PZ, Shaw JE (2020). Young-onset type 2 diabetes mellitus - implications for morbidity and mortality. Nat Rev Endocrinol.

[46] Sas DJ (2011). An update on the changing epidemiology and metabolic risk factors in pediatric kidney stone disease. Clin J Am Soc Nephrol.

[47] Novak TE, Lakshmanan Y, Trock BJ, Gearhart JP, Matlaga BR (2009). Sex prevalence of pediatric kidney stone disease in the United States: an epidemiologic investigation. Urology.

[48] Kirejczyk JK, Porowski T, Konstantynowicz J, Kozerska A, Nazarkiewicz A, Hoppe B (2014). Urinary citrate excretion in healthy children depends on age and gender. Pediatr Nephrol.

[49] Paik JM, Patorno E, Zhuo M, Bessette LG, York C, Gautam N (2022). Accuracy of identifying diagnosis of moderate to severe chronic kidney disease in administrative claims data. Pharmacoepidemiol Drug Saf.

