# Correction: Childhood diabetes mellitus and early-onset kidney diseases later in life: a nationwide population-based matched cohort study

**DOI:** 10.1186/s12916-023-02913-8

**Published:** 2023-06-01

**Authors:** Jiahong Sun, Ce Wang, Min Zhao, Priscilla M. Y. Lee, Bo Xi, Yongfu Yu, Jiong Li

**Affiliations:** 1 Department of Epidemiology, School of Public Health, Qilu Hospital, Cheeloo College of Medicine, Shandong University, 44 Wen Hua Xi Road, Jinan, 250012 Shandong China; 2 Department of Biostatistics, School of Public Health, and The Key Laboratory of Public Health Safety of Ministry of Education, Fudan University, 130 Dong’an, Shanghai, 200032 China; 3 Department of Nutrition and Food Hygiene, Cheeloo College of Medicine, Shandong University, Jinan, Shandong China; 4 Department of Clinical Medicine-Department of Clinical Epidemiology, Aarhus University Hospital, Aarhus, Denmark; 5 Shanghai Institute of Infectious Disease and Biosecurity, Shanghai, China

**Correction: BMC Med 20, 428 (2022)**

**https://doi.org/10.1186/s12916-022-02634-4**

The original article [1] contained inaccuracies in the Acknowledgements, Funding, and Availability of Data and Materials sections which have all been amended.

## References

[1] Sun J (2022). Childhood diabetes mellitus and early-onset kidney diseases later in life: a nationwide population-based matched cohort study. BMC Med.

