# Supplementary material for: Childhood diabetes mellitus and early-onset kidney diseases later in life: a nationwide population-based matched cohort study
Source: BMC Med. 2022 Nov 8;20:428. doi: 10.1186/s12916-022-02634-4 (PMC9641804; doi:10.1186/s12916-022-02634-4)
Supplement: Supplementary file 1 — Additional file 1: Supplementary results. Fig. S1. Flow chartof inclusion/exclusion of participants in this study. Fig. S2. Cumulative incidence of overall early-onset kidneydiseases later in life among children by age of diagnosis of type 1 diabetesmellitus (0-5, 6-12, and 13-17 years). Fig. S3. Cumulative incidence of overall early-onset kidney diseases later inlife among children by age of diagnosis of type 2 diabetes mellitus (0-5, 6-12,and 13-17 years). Table S1. International Classification of Diseases codes for diabetes mellitus and kidneydisease. Table S2. Associations ofchildhood diabetes with overall and type-specific early-onset kidney diseaseslater in life stratified for sex. TableS3. Associations of childhood diabetes with overall and type-specificearly-onset kidney diseases later in life stratified for periods of birth. Table S4. Associations of childhooddiabetes with overall and type-specific early-onset kidney diseases later inlife stratified for diagnosed age. TableS5. Associations of childhood diabetes with overall and type-specificearly-onset kidney disease later in life stratified for diabetes duration. Table S6. Associations of childhooddiabetes with overall and type-specific early-onset kidney disease later inlife stratified for number of diabetic complications. Table S7. Associations of childhood diabetes with overall andtype-specific early-onset kidney diseases later in life using a sibling design.Table S8. Associations of childhooddiabetes with overall and type-specific early-onset kidney diseases later inlife after excluding participants with one, three, or five years of diabetesduration. Table S9. Associations ofchildhood diabetes with overall and type-specific early-onset kidney diseaselater in life after exclusion of those without the use of hypoglycemic drugs. Table S10. Associations of childhooddiabetes with overall and type-specific early-onset kidney diseases later inlife after further adjustment for the use of nephroprotective thera [file 12916_2022_2634_MOESM1_ESM.doc]

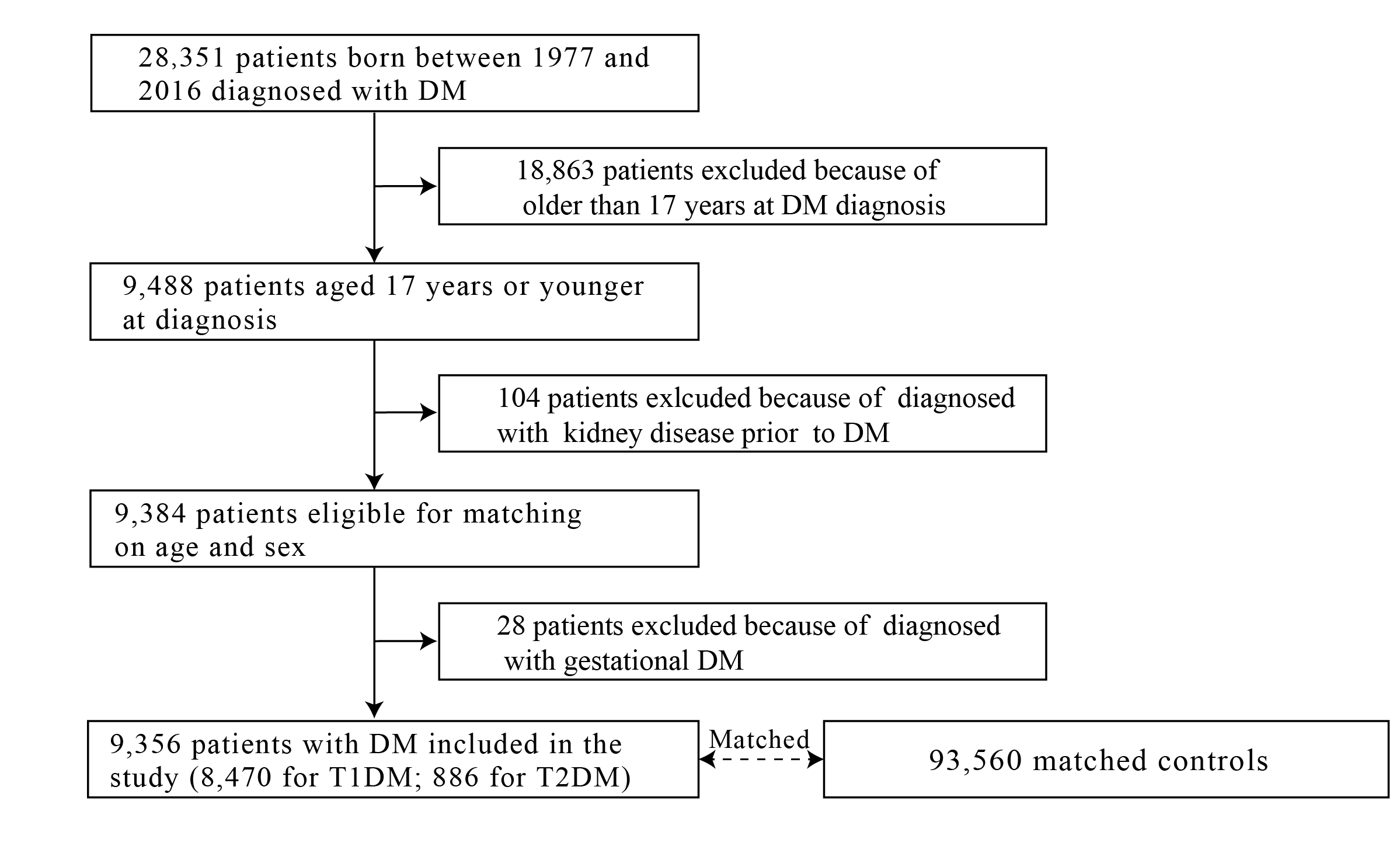


**Fig S1.** Flow chart of inclusion/exclusion of participants in this study


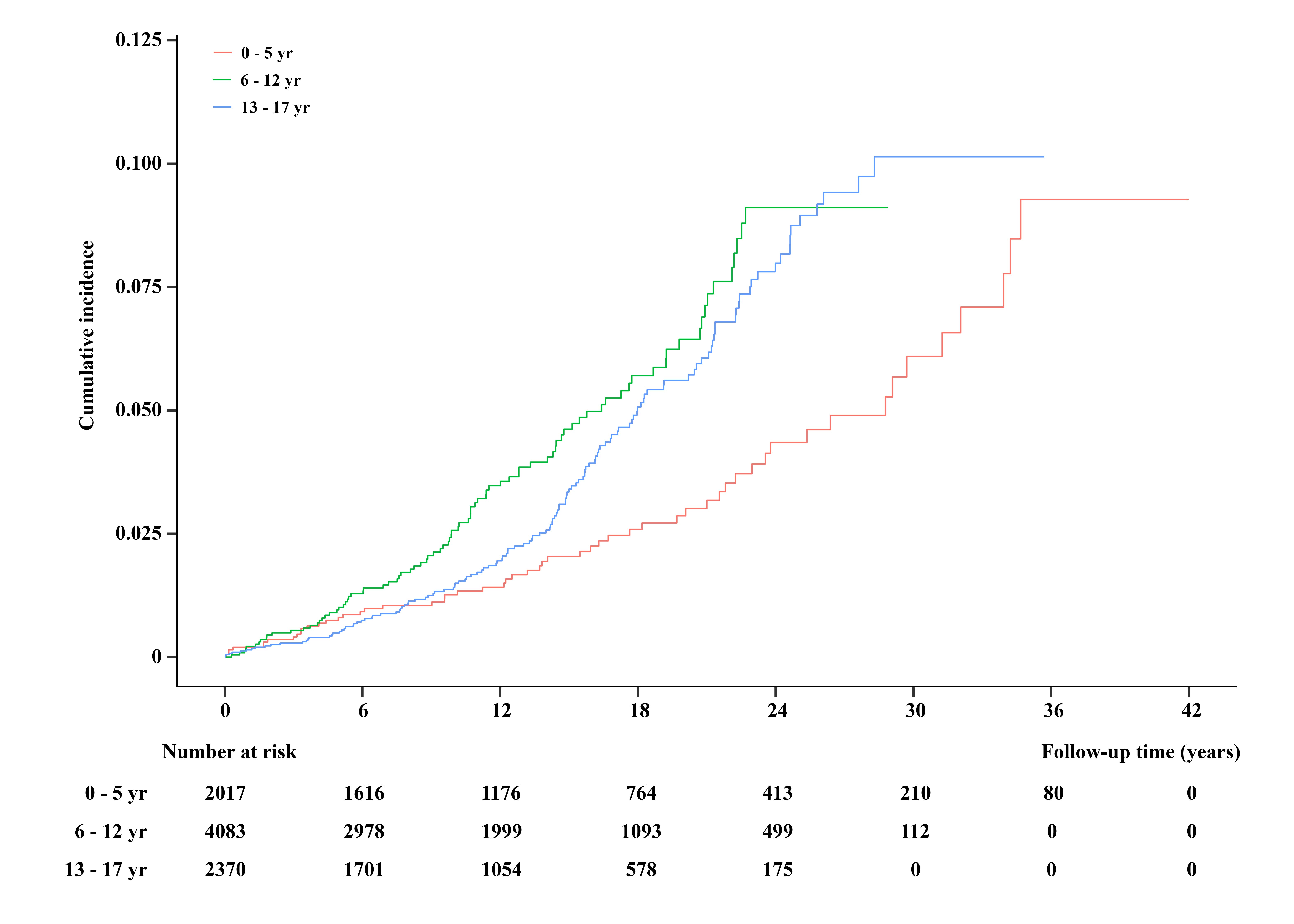


**Fig S2****.** Cumulative incidence of overall early-onset kidney diseases later in life among children by age of diagnosis of type 1 diabetes mellitus (0-5, 6-12, and 13-17 years)


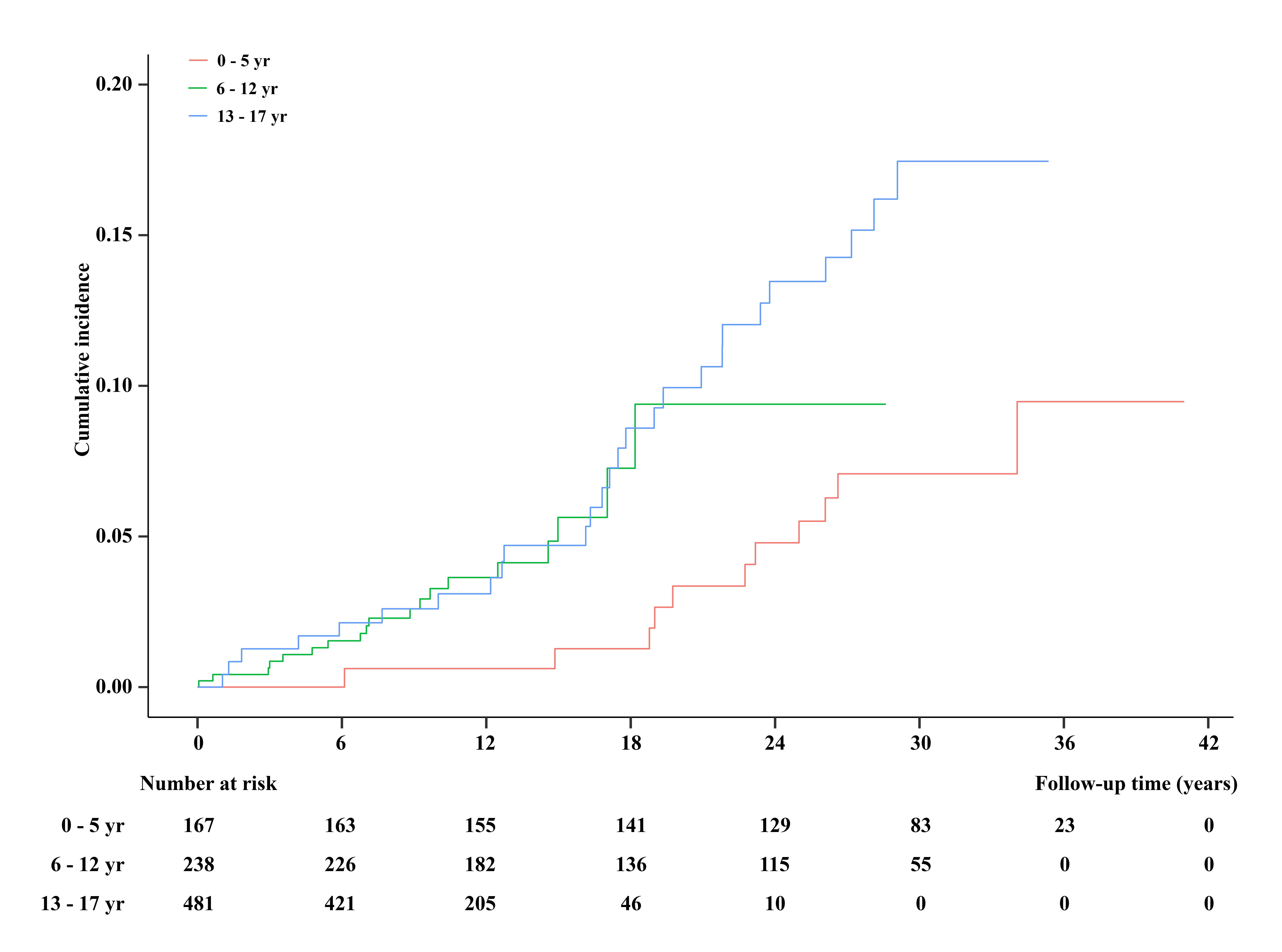


**Fig S3.** Cumulative incidence of overall early-onset kidney diseases later in life among children by age of diagnosis of type 2 diabetes mellitus (0-5, 6-12, and 13-17 years)

**Table S1.** International Classification of Diseases codes for diabetes mellitus and kidney disease

|  | **ICD code** | **Procedure/Surgery Codes** | **ATC** |
| --- | --- | --- | --- |
| **DM** |  |  |  |
| ***T1DM*** |  |  |  |
| T1DM without complications | ICD-10 | E10-E10.9, O24.0 | A10A |
| T1DM with coma | ICD-8; ICD-10 | ICD-8: 249.06, 249.07; ICD-10: E10.0 |  |
| T1DM with ketoacidosis | ICD-10 | E10.1 |  |
| T1DM with renal complications | ICD-8; ICD-10 | ICD-8: 249.02; ICD-10: E10.2 |  |
| T1DM with ophthalmic complications | ICD-8; ICD-10 | ICD-8: 249.02; ICD-10: E10.3 |  |
| T1DM with neurological complications | ICD-8; ICD-10 | ICD-8: 249.03; ICD-10: E10.4 |  |
| T1DM with peripheral circulatory complications | ICD-8; ICD-10 | ICD-8: 249.04, 249.05; ICD-10: E10.5 |  |
| T1DM with other specific complications | ICD-8; ICD-10 | ICD-8: 249.06, 249.07, 249.08; ICD-10: E10.6, E10.8 |  |
| T1DM with multiple complications | ICD-10 | E10.7 |  |
| ***T2DM*** |  |  | A10B |
| T2DM without complications | ICD-10 | E11-E11.9, O24.9 |  |
| T2DM with coma | ICD-8; ICD-10 | ICD-8: 250.06, 250.07; ICD-10: E11.0 |  |
| T2DM with ketoacidosis | ICD-10 | E11.1 |  |
| T2DM with renal complications | ICD-8; ICD-10 | ICD-8: 250.02; ICD-10: E11.2 |  |
| T2DM with ophthalmic complications | ICD-8; ICD-10 | ICD-8: 250.01; ICD-10: E11.3 |  |
| T2DM neurological complications | ICD-8; ICD-10 | ICD-8: 250.03; ICD-10: E11.4 |  |
| T2DM peripheral circulatory complications | ICD-8; ICD-10 | ICD-8: 250.04, 250.05; ICD-10: E11.5 |  |
| T2DM with other specific complications | ICD-8; ICD-10 | ICD-8: 250.06, 250.07, , 250.08; ICD-10: E11.6, E11.8 |  |
| T2DM with multiple complications | ICD-10 | E11.7 |  |
| **Specific kidney disease** |  |  |  |
| Glomerular diseases | ICD-10 | N00-N08 |  |
| Renal tubulo-interstitial diseases | ICD-10 | N10-N16 |  |
| Renal failure | ICD-10 | N17-N19 |  |
| Urolithiasis | ICD-10 | N20-N23 |  |
| Atherosclerosis of renal artery | ICD-10 | I70.1 |  |
| Arterial embolism and thrombosis of renal artery | ICD-8 | 444.3 |  |
| Renal glycosuria | ICD-10 | E74.8 |  |
| Hypertensive renal disease | ICD-10 | I12 |  |
| Hypertensive heart and renal disease | ICD-10 | I13 |  |
| Hepatorenal syndrome | ICD-10 | K76.7 |  |
| Hemorrhagic pulmonary renal syndrome | ICD-10 | M31.0 |  |
| Extrarenal uraemia | ICD-10 | R39.2 |  |
| Malignant neoplasm of kidney | ICD-8; ICD-10 | ICD-8: 189.0-189.1; ICD-10: C64-C65 |  |
| Benign neoplasm of kidney | ICD-8; ICD-10 | ICD-8:223.0-223.1; ICD-10: C30.0-C30.1 |  |
| Injury of renal blood vessels | ICD-10 | S35.4 |  |
| Injury of kidney | ICD-10 | S37.0 |  |
| Other disorders of kidney and ureter | ICD-10 | N25-N29 |  |
| ICD: International Classification of Disease; ATC: Anatomical Therapeutic Chemical | | |  |

**Table S2.** Associations of childhood diabetes with overall and type-specific early-onset kidney diseases later in life stratified for sex

| **Kidney disease** | **No (%) of kidney disease in exposed and unexposed** | **Rate per 1000 person-years in exposed and unexposed** | **Crude HR**  **(95% CI)** | **Adjusted HR**  **(95% CI)** |
| --- | --- | --- | --- | --- |
| **Male** |  |  |  |  |
| Any kidney disease | 121(2.48)/522(1.07) | 18.21/7.72 | 2.38(1.95-2.90) | 2.38(2.13-2.66) |
| Glomerular diseases | 29(0.59)/86(0.18) | 4.36/1.27 | 3.33(2.19-5.07) | 3.39(2.66-4.33) |
| Renal tubulo-interstitial diseases | 27(0.55)/112(0.23) | 4.06/1.66 | 2.47(1.62-3.77) | 2.45(1.93-3.10) |
| Renal failure | 52(1.06)/65(0.13) | 7.82/0.96 | 8.10(5.61-11.7) | 8.13(6.28-10.52) |
| Urolithiasis | 23(0.47)/257(0.53) | 3.46/3.80 | 0.93(0.61-1.42) | 0.92(0.71-1.19) |
| Injury of kidney | 6(0.12)/44(0.09) | 0.90/0.65 | 1.46(0.62-3.43) | 1.43(0.89-2.31) |
| Other disorders of kidney and ureter | 19(0.39)/47(0.10) | 2.86/0.70 | 4.16(2.44-7.12) | 4.21(3.03-5.85) |
| **Female** |  |  |  |  |
| Any kidney disease | 219(4.90)/854(1.91) | 36.6/13.89 | 2.64(2.27-3.06) | 2.65(2.43-2.88) |
| Glomerular diseases | 28(0.63)/75(0.17) | 4.68/1.22 | 3.88(2.50-6.00) | 3.78(2.88-4.95) |
| Renal tubulo-interstitial diseases | 139(3.11)/507(1.13) | 23.21/8.25 | 2.81(2.33-3.40) | 2.80(2.51-3.12) |
| Renal failure | 48(1.07)/50(0.11) | 8.01/0.81 | 10.67(7.09-16.07) | 10.61(7.79-14.44) |
| Urolithiasis | 42(0.94)/234(0.52) | 7.02/3.81 | 1.86(1.34-2.59) | 1.93(1.61-2.31) |
| Injury of kidney | — | — | — | — |
| Other disorders of kidney and ureter | 22(0.49)/42(0.09) | 3.68/0.68 | 5.31(3.16-8.92) | 5.45(3.92-7.59) |

Stratified Cox proportional regression analyses with inverse probability of treatment weighting based on propensity score were adjusted for birth weight, preterm birth, parity, singleton status, maternal residence, maternal marital status, maternal and paternal education level, and maternal and paternal history of diabetes.

HR, hazard ratio; CI, confidence interval

**Table S3.** Associations of childhood diabetes with overall and type-specific early-onset kidney diseases later in life stratified for periods of birth

| **Kidney disease** | **1977~1985** | | | |  | **1986~2016** | | | |
| --- | --- | --- | --- | --- | --- | --- | --- | --- | --- |
|  | **No (%) of kidney disease in exposed and unexposed** | **Rate per 1000 person-years in exposed and unexposed** | **Crude HR**  **(95% CI)** | **Adjusted HR**  **(95% CI)** |  | **No (%) of kidney disease in exposed and unexposed** | **Rate per 1000 person-years in exposed and unexposed** | **Crude HR**  **(95% CI)** | **Adjusted HR**  **(95% CI)** |
| Any kidney disease | 176(9.58)/596(3.24) | 37.87/12.41 | 3.05(2.58-3.62) | 3.03(2.74-3.34) |  | 164(2.18)/780(1.04) | 20.55/9.63 | 2.15(1.82-2.55) | 2.16(1.97-2.38) |
| Glomerular diseases | 37(2.01)/56(0.30) | 7.95/1.17 | 6.82(4.48-10.39) | 6.77(5.06-9.05) |  | 20(0.27)/105(0.14) | 2.51/1.3 | 1.91(1.18-3.08) | 1.88(1.44-2.47) |
| Renal tubulo-interstitial diseases | 69(3.75)/227(1.24) | 14.84/4.73 | 3.15(2.40-4.13) | 3.15(2.69-3.69) |  | 97(1.29)/392(0.52) | 12.15/4.84 | 2.53(2.02-3.16) | 2.50(2.20-2.84) |
| Renal failure | 77(4.19)/49(0.27) | 16.54/1.02 | 16.60(11.49-23.98) | 16.50(12.22-22.28) |  | 23(0.31)/66(0.09) | 2.88/0.81 | 3.69(2.29-5.96) | 3.73(2.81-4.96) |
| Urolithiasis | 33(1.80)/276(1.50) | 7.10/5.75 | 1.25(0.87-1.80) | 1.21(0.98-1.50) |  | 32(0.43)/215(0.29) | 4.01/2.65 | 1.52(1.05-2.21) | 1.59(1.30-1.95) |
| Injury of kidney | — | — | — | — |  | — | — | — | — |
| Other disorders of kidney and ureter | 25(1.36)/40(0.22) | 5.37/0.83 | 6.28(3.80-10.38) | 6.13(4.35-8.63) |  | 16(0.21)/49(0.07) | 2/0.6 | 3.39(1.92-5.98) | 3.56(2.57-4.94) |

Stratified Cox proportional regression analyses with inverse probability of treatment weighting based on propensity score were adjusted for birth weight, preterm birth, parity, singleton status, maternal residence, maternal marital status, maternal and paternal education level, and maternal and paternal history of diabetes.

HR, hazard ratio; CI, confidence interval

**Table S4.** Associations of childhood diabetes with overall and type-specific early-onset kidney diseases later in life stratified for diagnosed age

| **Kidney disease** | **0~5 years** | | | | **6~12 years** | | | | **13~17 years** | | | |
| --- | --- | --- | --- | --- | --- | --- | --- | --- | --- | --- | --- | --- |
|  | **No (%) of kidney disease in exposed and unexposed** | **Rate per 1000 person-**  **years in exposed and unexposed** | **Crude HR (95% CI)** | **Adjusted HR (95% CI)** | **No (%) of kidney disease in exposed and unexposed** | **Rate per 1000 person-**  **years in exposed and unexposed** | **Crude HR (95% CI)** | **Adjusted HR (95% CI)** | **No (%) of kidney disease in exposed and unexposed** | **Rate per 1000 person-**  **years in exposed and unexposed** | **Crude HR (95% CI)** | **Adjusted HR (95% CI)** |
| Ovearll kidney disease | 67(3.07)/ 347(1.59) | 18.56/  9.46 | 1.96 ( 1.51-2.55) | 1.93 ( 1.66-2.24) | 165(3.82)/ 624(1.44) | 28.97/  10.71 | 2.75 ( 2.31-3.27) | 2.76 ( 2.50-3.04) | 108(3.79)/ 405(1.42) | 32.51/  11.87 | 2.72 ( 2.20-3.37) | 2.75 ( 2.43-3.10) |
| Glomerular diseases | 9(0.41)/ 57(0.26) | 2.49/  1.55 | 1.60 ( 0.79-3.23) | 1.46 ( 0.95-2.24) | 30(0.69)/ 66(0.15) | 5.27/  1.13 | 4.65 ( 3.01-7.18) | 4.69 ( 3.58-6.15) | 18(0.63)/ 38(0.13) | 5.41/  1.11 | 4.70 ( 2.68-8.24) | 4.60  ( 3.21-6.60) |
| Renal tubulo-interstitial diseases | 38(1.74)/ 167(0.76) | 10.52/  4.55 | 2.31 ( 1.62-3.29) | 2.25 ( 1.83-2.76) | 80(1.85)/ 283(0.65) | 14.04/  4.86 | 2.92 ( 2.28-3.75) | 2.94 ( 2.55-3.39) | 48(1.68)/ 169(0.59) | 14.44/  4.95 | 2.90  ( 2.10-4.01) | 2.93 ( 2.43-3.52) |
| Renal failure | 10(0.46)/ 28(0.13) | 2.77/  0.76 | 3.76 ( 1.81-7.79) | 3.78 ( 2.44-5.86) | 56(1.30)/ 51(0.12) | 9.82/  0.88 | 11.91 ( 8.06-17.59) | 11.71 ( 8.66-15.83) | 34(1.19)/ 36(0.13) | 10.22/  1.05 | 9.56 ( 5.96-15.33) | 9.65 ( 6.84-13.61) |
| Urolithiasis | 16(0.73)/ 98(0.45) | 4.43/  2.67 | 1.65 ( 0.97-2.81) | 1.57 ( 1.15-2.16) | 28(0.65)/ 216(0.50) | 4.92/  3.71 | 1.37 ( 0.92-2.03) | 1.41 ( 1.13-1.75) | 21(0.74)/ 177(0.62) | 6.32/  5.19 | 1.22 ( 0.78-1.92) | 1.28 ( 0.99-1.64) |
| Injury of kidney | — | — | — | — | — | — | — | — | — | — | — | — |
| Other disorders of kidney and ureter | 7(0.32)/ 27(0.12) | 1.94/  0.74 | 2.58 ( 1.12-5.92) | 2.87 ( 1.82-4.52) | 22(0.51)/ 46(0.11) | 3.86/  0.79 | 4.8  ( 2.88-8.00) | 4.75 ( 3.44-6.56) | 12(0.42)/ 16(0.06) | 3.61/  0.47 | 8.54 ( 3.95-18.46) | 8.82 ( 5.14-15.14) |

Stratified Cox proportional regression analyses with inverse probability of treatment weighting based on propensity score were adjusted for birth weight, preterm birth, parity, singleton status, maternal residence, maternal marital status, maternal and paternal education level, and maternal and paternal history of diabetes.

HR, hazard ratio; CI, confidence interval

**Table S5.** Associations of childhood diabetes with overall and type-specific early-onset kidney disease later in life stratified for diabetes duration

| **Kidney disease** | **0~10 year** | | | |  | **10~20 year** | | | |  | **20~ year** | | | |
| --- | --- | --- | --- | --- | --- | --- | --- | --- | --- | --- | --- | --- | --- | --- |
|  | **No (%) of kidney disease in exposed and unexposed** | **Rate per 1000 person-years in exposed and unexposed** | **Crude HR (95% CI)** | **Adjusted HR(95% CI)** |  | **No (%) of kidney disease in exposed and unexposed** | **Rate per 1000 person-**  **years in exposed and unexposed** | **Crude HR (95% CI)** | **Adjusted HR (95% CI)** |  | **No (%) of kidney disease in exposed and unexposed** | **Rate per 1000 person-**  **years in exposed and unexposed** | **Crude HR (95% CI)** | **Adjusted HR(95% CI)** |
| Any kidney disease | 133(1.42) /661(0.71) | 17.81/  8.78 | 2.04 (1.69-2.45) | 2.07 (1.86-2.29) |  | 137(2.49) /485(0.89) | 36.42 /12.86 | 2.83 (2.34-3.43) | 2.80 (2.51-3.13) |  | 70(3.13) /199(0.92) | 50.10/  14.25 | 3.47 (2.64-4.56) | 3.43 (2.90-4.05) |
| Glomerular diseases | 20(0.21) /105(0.11) | 2.68/  1.40 | 1.89 (1.17-3.05) | 1.80 (1.36-2.38) |  | 22(0.40) /40(0.07) | 5.84/  1.06 | 5.38 (3.2-9.06) | 5.39 (3.85-7.54) |  | 15(0.67) /14(0.06) | 10.73/  1.00 | 12.07 (5.65-25.78) | 12.60 (7.02-22.61) |
| Renal tubulo-interstitial diseases | 73(0.78) /331(0.35) | 9.77/  4.40 | 2.24 (1.73-2.88) | 2.28 (1.98-2.62) |  | 67(1.22) /205(0.38) | 17.8/  5.43 | 3.31 (2.51-4.36) | 3.24 (2.75-3.82) |  | 26(1.16) /73(0.34) | 18.6/  5.22 | 3.51 (2.24-5.5) | 3.29 (2.49-4.36) |
| Renal failure | 18(0.19) /47(0.05) | 2.41/  0.62 | 3.91 (2.27-6.75) | 3.98 (2.87-5.52) |  | 50(0.91) /44(0.08) | 13.27/  1.17 | 11.07 (7.38-16.6) | 11.01 (8.10-14.97) |  | 32(1.43) /18(0.08) | 22.86/  1.28 | 18.19 (10.10-32.76) | 18.09 (11.09-29.5) |
| Urolithiasis | 18(0.19) /151(0.16) | 2.41/  2.01 | 1.21 (0.74-1.97) | 1.26 (0.96-1.66) |  | 27(0.49) /210(0.39) | 7.17/  5.56 | 1.29 (0.87-1.93) | 1.32 (1.05-1.66) |  | 20(0.89) /113(0.52) | 14.30/  8.08 | 1.73 (1.08-2.79) | 1.63 (1.23-2.16) |
| Injury of kidney | — | — | — | — |  | — | — | — | — |  | — | — | — | — |
| Other disorders of kidney and ureter | 15(0.16) /48(0.05) | 2.01/  0.64 | 3.26 (1.82-5.83) | 3.39 (2.44-4.73) |  | 19(0.35) /25(0.05) | 5.05/  0.66 | 7.42 (4.08-13.47) | 7.59 (5.00-11.52) |  | 7(0.31) /15(0.07) | 5.01/  1.07 | 4.60 (1.87-11.28) | 4.60 (2.63-8.03) |

Stratified Cox proportional regression analyses with inverse probability of treatment weighting based on propensity score were adjusted for birth weight, preterm birth, parity, singleton status, maternal residence, maternal marital status, maternal and paternal education level, and maternal and paternal history of diabetes.

HR, hazard ratio; CI, confidence interval

**Table S6.** Associations of childhood diabetes with overall and type-specific early-onset kidney disease later in life stratified for number of diabetic complications

| **Kidney disease** | **0 complication** | | | |  | **≥1 complications** | | | |
| --- | --- | --- | --- | --- | --- | --- | --- | --- | --- |
|  | **No (%) of kidney disease in exposed and unexposed** | **Rate per 1000 person-years in exposed and unexposed** | **Crude HR** | **Adjusted HR**  **(95% CI)** |  | **No (%) of kidney disease in exposed and unexposed** | **Rate per 1000 person-years in exposed and unexposed** | **Crude HR** | **Adjusted HR (95% CI)** |
| Any kidney disease | 69(1.67)/455(1.1) | 16.3/10.58 | 1.53 (1.19-1.97) | 1.58(1.37-1.82) |  | 271(5.18)/921(1.76) | 32.28/10.7 | 3.05(2.66-3.5) | 3.02(2.79-3.27) |
| Glomerular diseases | 7(0.17)/61(0.15) | 1.65/1.42 | 1.13 (0.52-2.47) | 1.24(0.81-1.88) |  | 50(0.96)/100(0.19) | 5.95/1.16 | 5.13(3.65-7.22) | 5.07(4.06-6.33) |
| Renal tubulo-interstitial diseases | 27(0.65)/211(0.51) | 6.38/4.91 | 1.29 (0.86-1.92) | 1.31(1.04-1.64) |  | 139(2.66)/408(0.78) | 16.55/4.74 | 3.53(2.91-4.29) | 3.49(3.11-3.92) |
| Renal failure | 19(0.46)/40(0.1) | 4.49/0.93 | 4.8 (2.77-8.3) | 4.96(3.52-6.98) |  | 81(1.55)/75(0.14) | 9.64/0.87 | 11.65(8.44-16.08) | 11.51(8.99-14.73) |
| Urolithiasis | 17(0.41)/143(0.35) | 4.02/3.32 | 1.22 (0.74-2.02) | 1.31(0.99-1.72) |  | 48(0.92)/348(0.67) | 5.71/4.04 | 1.44(1.06-1.95) | 1.41(1.19-1.68) |
| Injury of kidney | NA | NA | NA | NA |  | NA | NA | NA | NA |
| Other disorders of kidney and ureter | 11(0.27)/27(0.07) | 2.6/0.63 | 4.05 (2.01-8.17) | 4.19(2.75-6.39) |  | 30(0.57)/62(0.12) | 3.57/0.72 | 5.01(3.23-7.78) | 5.05(3.82-6.68) |

Stratified Cox proportional regression analyses with inverse probability of treatment weighting based on propensity score were adjusted for birth weight, preterm birth, parity, singleton status, maternal residence, maternal marital status, maternal and paternal education level, and maternal and paternal history of diabetes.

HR, hazard ratio; CI, confidence interval

**Table S7.** Associations of childhood diabetes with overall and type-specific early-onset kidney diseases later in life using a sibling design

| **Kidney disease** | **No (%) of kidney disease in exposed and unexposed** | **Rate per 1000 person-years in exposed and unexposed** | **Crude HR (95% CI)** | **Adjusted HR (95% CI)** |
| --- | --- | --- | --- | --- |
| Any kidney disease | 213(3.02)/129(1.26) | 24.87/10.55 | 2.37(1.88-2.98) | 2.39(2.00-2.87) |
| Glomerular diseases | 34(0.48)/17(0.17) | 3.97/1.39 | 2.64(1.45-4.83) | 2.78(1.7-4.54) |
| Renal tubulo-interstitial diseases | 109(1.55)/65(0.63) | 12.73/5.31 | 2.34(1.70-3.23) | 2.36(1.83-3.04) |
| Renal failure | 47(0.67)/17(0.17) | 5.48/1.39 | 4.66(2.52-8.61) | 4.88(2.89-8.24) |
| Urolithiasis | 40(0.57)/46(0.45) | 4.67/3.76 | 1.19(0.76-1.85) | 1.21(0.87-1.67) |
| Injury of kidney | 6(0.09)/7(0.07) | 0.70/0.57 | 1.24(0.41-3.79) | 1.31(0.57-3.03) |
| Other disorders of kidney and ureter | 21(0.30)/8(0.08) | 2.45/0.65 | 4.08(1.77-9.42) | 4.04(1.97-8.28) |

Stratified Cox proportional regression analyses with inverse probability of treatment weighting based on propensity score were adjusted for birth weight, preterm birth, parity,

singleton status, maternal residence, maternal marital status, maternal and paternal education level, and maternal and paternal history of diabetes.

HR, hazard ratio; CI, confidence interval; DM

**Table S8.** Associations of childhood diabetes with overall and type-specific early-onset kidney diseases later in life after excluding participants with one, three, or five years of diabetes duration

| **Kidney disease** | **Excluding one year duration** | | | |  | **Excluding three year duration** | | | |  | **Excluding five year duration** | | | |
| --- | --- | --- | --- | --- | --- | --- | --- | --- | --- | --- | --- | --- | --- | --- |
|  | **No (%) of kidney disease in exposed and unexposed** | **Rate per 1000 person-**  **years in exposed and unexposed** | **Crude HR (95% CI)** | **Adjusted HR** **(95% CI)** |  | **No (%) of kidney disease in exposed and unexposed** | **Rate per 1000 person-**  **years in exposed and unexposed** | **Crude HR (95% CI)** | **Adjusted HR** **(95% CI)** |  | **No (%) of kidney disease in exposed and unexposed** | **Rate per 1000 person-**  **years in exposed and unexposed** | **Crude HR (95% CI)** | **Adjusted HR (95% CI)** |
| Any kidney disease | 321(3.59) /1307(1.46) | 24.20/  9.22 | 2.51 ( 2.22-2.84) | 2.52 ( 2.35-2.70) |  | 293(3.67) /1136(1.42) | 22.71/  8.41 | 2.64  ( 2.32-3.00) | 2.64 ( 2.45-2.84) |  | 262(3.70) /986(1.39) | 22.71/  8.41 | 2.74 ( 2.39-3.14) | 2.74 ( 2.54-2.97) |
| Glomerular diseases | 55(0.62) /142(0.16) | 4.21/  0.87 | 3.92 ( 2.87-5.36) | 3.94 ( 3.26-4.76) |  | 51(0.64) /107(0.13) | 4.16/  0.73 | 4.84 ( 3.46-6.77) | 4.85 ( 3.93-5.99) |  | 48(0.68) /86(0.12) | 4.16/  0.73 | 5.71 ( 4.00-8.14) | 5.75 ( 4.56-7.25) |
| Renal tubulo-  Interstitial diseases | 157(1.76) /585(0.65) | 11.56/  3.96 | 2.74 ( 2.30-3.27) | 2.73 ( 2.47-3.02) |  | 140(1.76) /488(0.61) | 10.57/  3.49 | 2.95 ( 2.44-3.56) | 2.93 ( 2.62-3.26) |  | 122(1.72) /409(0.58) | 10.57/  3.49 | 3.08 ( 2.52-3.78) | 3.07 ( 2.73-3.46) |
| Renal failure | 96(1.07) /109(0.12) | 7.59/  0.76 | 9.22 ( 6.98-12.18) | 9.18 ( 7.49-11.25) |  | 92(1.15) /93(0.12) | 7.53/  0.69 | 10.37 ( 7.74-13.91) | 10.33 ( 8.30-12.86) |  | 87(1.23) /81(0.11) | 7.53/  0.69 | 11.25 ( 8.27-15.30) | 11.17 ( 8.84-14.11) |
| Urolithiasis | 60(0.67) /482(0.54) | 4.71/  3.65 | 1.29 ( 0.98-1.69) | 1.31 ( 1.12-1.52) |  | 57(0.71) /449(0.56) | 4.68/  3.60 | 1.30 ( 0.99-1.72) | 1.32 ( 1.13-1.55) |  | 54(0.76) /422(0.60) | 4.68/  3.60 | 1.32 ( 0.99-1.75) | 1.33 ( 1.13-1.57) |
| Injury of kidney | 7(0.08) /64(0.07) | 0.5/  0.48 | 1.11 ( 0.51-2.43) | 1.10  ( 0.70-1.74) |  | 6(0.08) /59(0.07) | 0.35/  0.40 | 1.04 ( 0.45-2.41) | 1.03 ( 0.62-1.69) |  | — | — | — | — |
| Other disorders of kidney and ureter | 34(0.38) /85(0.10) | 2.72/  0.57 | 4.09 ( 2.74-6.10) | 4.16 ( 3.26-5.31) |  | 33(0.41) /70(0.09) | 2.69/  0.45 | 4.79 ( 3.16-7.25) | 4.90 ( 3.77-6.35) |  | 31(0.44) /53(0.07) | 2.69/  0.45 | 6.00 ( 3.84-9.37) | 6.17 ( 4.60-8.28) |

Stratified Cox proportional regression analyses with inverse probability of treatment weighting based on propensity score were adjusted for birth weight, preterm birth, parity, singleton status, maternal residence, maternal marital status, maternal and paternal education level, and maternal and paternal history of diabetes.

HR, hazard ratio; CI, confidence interval

**Table S9.** Associations of childhood diabetes with overall and type-specific early-onset kidney disease later in life after exclusion of those without the use of hypoglycemic drugs

| **Kidney disease** | **No (%) of kidney disease in exposed and unexposed** | **Rate per 1000 person-years in exposed and unexposed** | **Crude HR** | **Adjusted HR (95% CI)** |
| --- | --- | --- | --- | --- |
| Any kidney disease | 308(3.62)/1198(1.41) | 27.31/10.40 | 2.65(2.33-3.00) | 2.60(2.46-2.84) |
| Glomerular diseases | 54(0.63)/138(0.16) | 4.79/1.20 | 3.98(2.90-5.45) | 3.98(3.29-4.83) |
| Renal tubulo-interstitial diseases | 156(1.83)/536(0.63) | 13.83/4.65 | 2.99(2.50-3.58) | 2.97(2.68-3.30) |
| Renal failure | 89(1.05)/97(0.11) | 7.88/0.84 | 9.58(7.15-12.83) | 9.51(7.67-11.79) |
| Urolithiasis | 58(0.68)/436(0.51) | 5.14/3.79 | 1.38(1.05-1.82) | 1.39(1.19-1.62) |
| Injury of kidney | 7(0.08)/63(0.07) | 0.62/0.55 | 1.16(0.53-2.53) | 1.16(0.73-1.83) |
| Other disorders of kidney and ureter | 34(0.40)/81(0.10) | 3.01/0.70 | 4.30(2.88-6.44) | 4.39(3.43-5.61) |

Stratified Cox proportional regression analyses with inverse probability of treatment weighting based on propensity score were adjusted for birth weight, preterm birth, parity, singleton status, maternal residence, maternal marital status, maternal and paternal education level, and maternal and paternal history of diabetes.

HR, hazard ratio; CI, confidence interval

**Table S10.** Associations of childhood diabetes with overall and type-specific early-onset kidney diseases later in life after further adjustment for the use of nephroprotective therapy

| **Kidney disease** | **No (%) of kidney disease in exposed and unexposed** | **Rate per 1000 person-years in exposed and unexposed** | **Crude HR (95% CI)** | **Adjusted HR (95% CI)** |
| --- | --- | --- | --- | --- |
| Any kidney disease | 340(3.63)/1376(1.47) | 26.93/10.66 | 2.54(2.25-2.86) | 2.44(2.27-2.62) |
| Glomerular diseases | 57(0.61)/161(0.17) | 4.51/1.25 | 3.58(2.64-4.84) | 3.30(2.71-4.02) |
| Renal tubulo-interstitial diseases | 166(1.77)/619(0.66) | 13.14/4.80 | 2.75(2.32-3.27) | 2.66(2.40-2.95) |
| Renal failure | 100(1.07)/115(0.12) | 7.91/0.89 | 9.16(6.97-12.03) | 8.72(7.11-10.70) |
| Urolithiasis | 65(0.69)491(0.52) | 5.15/3.80 | 1.37(1.06-1.78) | 1.35(1.16-1.58) |
| Injury of kidney | 8(0.09)/72(0.08) | 0.63/0.56 | 1.15(0.55-2.38) | 1.10(0.70-1.73) |
| Other disorders of kidney and ureter | 41(0.44)89(0.10) | 3.25/0.69 | 4.71(3.25-6.83) | 4.60(3.61-5.87) |

Stratified Cox proportional regression analyses with inverse probability of treatment weighting based on propensity score were adjusted for birth weight, preterm birth, parity, singleton status, maternal residence, maternal marital status, maternal and paternal education level, maternal and paternal history of diabetes, and the use of nephroprotective therapy.

HR, hazard ratio; CI, confidence interval
